# A qualitative process evaluation of electronic session-by-session outcome measurement in child and adolescent mental health services

**DOI:** 10.1186/1471-244X-14-113

**Published:** 2014-04-15

**Authors:** Charlotte L Hall, John Taylor, Maria Moldavsky, Michael Marriott, Sarah Pass, Karen Newell, Robert Goodman, Kapil Sayal, Chris Hollis

**Affiliations:** 1 CLAHRC-NDL, University of Nottingham, Nottingham, UK; 2 Nottinghamshire Healthcare NHS Trust, Nottingham, UK; 3 King's College London, Institute of Psychiatry, London, UK; 4 Developmental Psychiatry, University of Nottingham, Queen’s Medical Centre, Nottingham, UK; 5 B07 Institute of Mental Health, University of Nottingham, Triumph Road, Nottingham NG7 2TU, UK

**Keywords:** Session by session, CAMHS, Qualitative evaluation, Parent, Clinician, Outcome measures

## Abstract

**Background:**

Regular monitoring of patient progress is important to assess the clinical effectiveness of an intervention. Recently, initiatives within UK child and adolescent mental health services (CAMHS) have advocated the use of session-by-session monitoring to continually evaluate the patient’s outcome throughout the course of the intervention. However, the feasibility and acceptability of such regular monitoring is unknown.

**Method:**

Semi-structured qualitative interviews were conducted with clinicians (*n* = 10), administrative staff (*n* = 8) and families (*n* = 15) who participated in a feasibility study of an electronic session-by-session outcome monitoring tool, (SxS), which is based on the Strengths and Difficulties Questionnaire (SDQ). This study took place in three CAMHS clinics in Nottinghamshire. The interview transcripts were thematically analysed.

**Results:**

We found clinicians accepted the need to complete outcome measures, particularly valuing those completed by the patient. However, there were some difficulties with engaging clinicians in this practice and in the training offered. Generally, patients were supportive of completing SxS in the waiting room prior to the clinic session and assistance with the process from administrative staff was seen to be a key factor. Clinicians and families found the feedback reports created from SxS to be helpful for tracking progress, facilitating communication and engagement, and as a point of reflection. The use of technology was considered positively, although some technological difficulties hindered the completion of SxS. Clinicians and families appreciated the brevity of SxS, but some were concerned that a short questionnaire could not adequately encapsulate the complexity of the patient’s issues.

**Conclusions:**

The findings show the need for appropriate infrastructure, mandatory training, and support to enable an effective system of session-by-session monitoring. Our findings indicate that clinicians, administrative staff and young people and their parents/carers would support regular monitoring if the system is easy to implement, with a standard ‘clinic-wide’ adoption of the procedure, and the resulting data are clinically useful.

## Background

In the NHS Outcomes Framework policy [1], the UK government highlighted the importance of assessing outcomes to enable measurement of the effectiveness of services. For child and adolescent mental health services (CAMHS), the National Service Framework (NSF) suggests that CAMHS interventions should be regularly monitored in order to improve clinical work and inform future service development [2]. The NSF specifically recognises the importance of measuring the patient’s perspective of outcome, including, where possible, the views of the young person, and notes the importance of administrative and clinical support to enable this process.

To support the use of outcome measures, the CAMHS Outcome Research Consortium (CORC; http://www.corc.uk.net) was created to develop a common suite of outcome measures and support services with the collection and analysis of anonymised outcome data. Despite this support, studies have noted little uptake of routine outcome measurement (ROM) within CAMHS in the UK [3,4], and particularly low rates of repeated use of outcome measures [3,5-7].

Despite ROM being valued for improving patient monitoring and outcome, aiding goal-setting, encouraging evidence-based practice, increasing patient input and improving clinicians ability to predict outcome in adult services [8-11], there are several factors inhibiting the use of outcome measures. Research has indicated a lack of time, resources, training and feedback from the measures to be fundamental barriers to their use [3,4], [12,13]. Additional concerns focus on data misuse, questionnaires being unable to capture complexity of issues and regular monitoring not fitting with all therapeutic approaches [11,12,14,15].

Less research has focused on patient perceptions of ROM. Two studies found that families attending CAMHS felt it was important for their progress to be tracked [16,17]. Families particularly noted the importance of having feedback from the measures they completed, being able to discuss the data with their clinician, and having brief measures that were holistic and easy to complete.

Since 2011, CORC have been commissioned by the Department of Health to support the analysis of outcome measurements collated through the Children and Young People’s Improving Access to Psychological Therapies (CYP-IAPT; http://www.IAPT.nhs.uk). The CYP-IAPT aspires to improve services for patients by routinely assessing their opinion on the quality and experience of services and specifically advocates the use of session-by-session monitoring to achieve this. Based on the experiences in adult therapeutic studies, the implementation of session-by-session monitoring in CAMHS may improve the completion of follow-up measures, help clinicians detect sudden large improvements [18] and lead to better patient outcome [9], however, little is known about the feasibility and acceptability of this system.

We piloted the use of a short electronic 8-item questionnaire, known as SxS (http://www.sdqinfo.org/SxS). Electronic based questionnaires may offer a particularly advantageous way of collecting ROM. Research has shown that electronic measures encourage people to answer more honestly [19], improve the effectiveness of the assessment [20], and offer the opportunity to present items in a ‘user friendly’ manner, which has been identified as a key point in improving their use in practice [16].

SxS is based upon the Strengths and Difficulties Questionnaire (SDQ) impact supplement [21], but also contains a question about improvement in symptoms and another about hope for the future. The measure is not symptom-specific and there are two versions; one for completion by the young person (11-17-years) and one for their parent/carer. The measure is designed to assess the young person and parent/carer’s perception of their progress since their last clinic appointment. SxS was completed on an iPad in the waiting room, prior to the clinic appointment. Young people and/or their parents/carers participating in the pilot were asked to complete SxS before every clinic session. A report graphing the young person’s progress was automatically generated with the intention of being discussed with the clinician during the appointment.

In order to assess the utility of SxS as a session-by-session measure, we sought to gain perceptions of feasibility and acceptability for healthcare professionals (HCPs), young people and parents, and administrative teams who were involved in the process. In the literature there is a notable absence of clinician and patient opinion on the use of outcome measures and to the best of our knowledge no research has assessed opinions on session-by-session measures within CAMHS.

## Method

### Participants

The pilot was conducted across three CAMHS teams in Nottinghamshire Healthcare NHS Trust (NHT). All individuals (HCPs, administrative staff, young people and parent/carers) who were involved in the pilot were invited by the researcher to participate in an interview about their experience of SxS. Ethical approval was granted by the local Research Ethics Committee and Research and Development Department of Nottinghamshire NHS Healthcare Trust. The research was conducted by the National Institute of Health Research (NIHR) Collaborations for Leadership in Applied Health Research and Care-Nottinghamshire, Derbyshire and Lincolnshire (CLAHRC-NDL).

*HCPs.* Ten out of the 13 (77%) HCPs that participated in SxS agreed to be interviewed. The interview sample consisted of 5 Clinical Psychologists, 2 Mental Health Nurses, 1 Nurse Prescriber, 1 Consultant Psychiatrist and 1 trainee Psychiatrist. Eight HCPs were female, two were male, with experience of working in CAMHS ranging from 1–11 years (*M* = 7.0 years, *SD* = 3.5).

#### *Young people and parents/carers*

Fifteen out of the 31 (48%) families who participated in SxS agreed to be interviewed. The young people were being treated for a range of diagnoses including Attention Deficit/Hyperactivity Disorder (ADHD), Autism Spectrum Disorder (ASD), Tourette’s Syndrome, Anxiety Disorders, Post-Traumatic Stress Disorders (PTSD), Eating Disorders and Depression. The young people ranged in age from 11–19 years (*M = 15* years, *SD* = 1.9), 8 were female, 7 were male. Young people and their parents were given a small inconvenience allowance for their participation. All participants were new to the service at the time of starting the pilot (case open for less than 2 months).

#### *Administrative Staff*

All 8 (100%) administration staff that participated in SxS agreed to be interviewed. The sample consisted of 4 general administrators, 3 medical secretaries and 1 administration manager. Administrative staff were given a small monthly inconvenience allowance for the duration of the SxS pilot. All administration staff were female, with experience of working in CAMHS administration ranging from 1 – 15 years (*M* = 5.6 years, *SD* = 5.0).

### Procedure

Prior to the interviews taking place, all participants were asked to read an information sheet outlining the process and signed a consent form.

All interviews were conducted by the lead researcher (CLH) and recorded on a dictaphone to aid subsequent transcription. Clinician and administrative staff interviews took place individually in their clinics. Interviews with the young people and their families were either conducted at the clinic or in their home.

The interviews were semi-structured and guided by separate interview schedules created for each of the participant groups (HCPs, administrative staff and young people/parents/carers; see Additional files 1, 2 and 3). The use of a semi-structured interview format allowed the researcher flexibility to ask additional questions based on the interviewees responses, and a set of prompt questions under each interview question were used to stimulate discussion if needed [22]. A series of prompts were utilised to stimulate responses if required. Topics of discussion included opinions on outcome measures, ease of implementation and completion of SxS, opinions on SxS questions and reports, and the future of SxS.

### Analysis

Audio recordings were anonymised and transcribed verbatim. All transcripts were first analysed inductively by the lead researcher (CLH) using the guidelines of Braun and Clarke [23]. Each coding unit was coded exclusively into just one category rather than into multiple categories as this approach creates clearly defined coding categories [24]. As validity and reliability of data interpretation are crucial to qualitative enquiry [25], inter-rater agreement was established by an independent researcher (KN). KN assigned themes to a random sample of 10% (166) of quotes from all the transcripts on the basis of the theme descriptions provided by the lead researcher. As the second-coder assigned 148 of the 166 quotes (89%) to the ‘correct’ theme, this demonstrated a high level of agreement with the coding decisions made by the lead researcher. Instances where there were inconsistencies between the two coders were resolved through discussion to reach consensus.

The researchers utilised an essentiality/realist paradigm [23] that sought to understand opinions on SxS as a session-by-session measure through the words of the participants, as opposed to the researchers’ co-created meaning. All codes came inductively from the data.

## Results and discussion

Five salient themes emerged from the data relating to clinicians’ general opinion of outcome measures, the SxS process, the helpfulness of SxS, attitudes towards technology and the SxS content. A summary of themes and their associated sub-themes is provided in Table 1.

**Table 1:** Summary of themes and sub-themes

**Main Theme**	**Subcthemes**
General opinion of outcome measures	
SxS process	Initiating SxS
	Time factors
	Timing & location for SxS
	Applicability of SxS
	Sustaining SxS
Helpfulness of SxS	Progress tracker
	Communication & engagement
	Improvements to reports
	Reflection
Attitudes towards technology	
SxS content	

### Theme 1 – general attitudes to outcome measures

Only HCPs were asked about their generic opinion of other outcome measures. Reasons reported by HCPs for using outcome measures included the need to inform clinical practice and to satisfy demands from managers. However, in support of previous research [3], clinicians often reported that they did not find outcome measures clinically useful, due to a lack of feedback.

“I do think they are important for the service… but I have to say I don't ever see any data coming back from them, so I don't think they are very useful in that sense, it would feel more useful if we were getting feedback from them” (HCP 4)

Consistent with previous findings [4], HCPs tended to value measures completed by the patient as more important than measures completed by themselves. It seemed that this was because it allowed the patient to have their voice heard and their opinion was less subject to bias.

“So I think it's usually helpful for families to be able to feel they have a voice and say what their experiences have been” (HCP 4)

“The disadvantage is, I guess, that with the clinician rated [measure], there is some subjectivity, when you're kind of calibrating someone that you are working with… there's potential for a kind of bias” (HCP 3)

Some HCPs commented that outcome measures such as the Health of the Nation Outcome Scales for Children and Adolescents (HoNOSCA; [26]), the Children’s Global Assessment Scale (C-GAS; [27]) and the Strengths and Difficulties Questionnaire (SDQ; [28]) were too generic to offer much clinically helpful information on the individual patient. This supported previous findings [4], [11,29] where these generic measures were not considered specific enough to detect changes related to a given disorder. Recently, Wolpert et al. [30] have also acknowledged the challenge for ROM in choosing measures specific enough to be clinically useful but general enough to allow comparisons across services.

In general, HCPs were supportive of using outcome measures as an adjunct to support clinical opinion, but some raised concerns about favouring outcome measures over clinical judgment [4,15].

“I think…. there is a danger in just focusing purely on outcome measures without any kind of interpretation and qualitative information” (HCP 2)

It is interesting to note that HCPs working within a CORC and CYP-IAPT adopted NHS Trust noted such concerns, despite these organisations firmly advocating that quantitative measures are intended to complement rather than replace clinical judgement [29,31-33].

### Theme 2 – the SxS process

All participants (HCPs, administration staff and patients) were asked about the SxS procedure.

#### *Initiating* SxS

In general, the administrative staff felt well informed of what would be expected of them, but had initial concerns about having to remember which patients were participating in SxS, handing out the iPad, and combining this new responsibility with existing demands.

“Knowing how busy our reception desk can get, when we've got everybody else's needs as well and then when you're taking on something else that was alien to you, it was nerve-wracking” (Admin 8)

However, in all cases they reported valuing the information and support provided by the research team and found that the process became easier over time.

“And we’ve got information packs, so it’s really straight forward and I think CH [researcher] helped… go through the pack and helped with everything” (Admin 2)

These findings indicate that if administrative staff feel fully informed and supported in how to administer ROM this can overcome any initial resistance to taking on extra responsibility. Furthermore, although the initial stages of implementing a new format of ROM are likely to be the most difficult [30,32], the process should improve once the routine is embedded in practice.

Families reported feeling happy to participate.

“Well [HCP] basically suggested to me and said I think it would be a good idea because, you could see how your progress is making, through using it and I was like oh yeah I'll try it, it's something new to try so” (Young Person 14)

HCPs’ reasons for participating in SxS included enjoying being involved in new ideas and wanting to improve the routine collection of outcome measures. The majority of HCPs reported feeling well prepared to participate in the process.

“Yeah I think the information that we had was pretty clear and concise and that I understood what was what, which was probably partly why I was fairly keen to do it, because it was quite clear” (HCP 4)

However, despite the study team providing several emails, flyers, attending clinic away days and team meetings and a ‘clinic champion’ being present at each site to provide on-site training, some HCPs reported that they were unclear about the SxS process.

“…Slightly bemused if I'm honest because I thought I think I should know about this and I don’t. And I think to be fair, I probably had had emails but I haven't really read them in detail” (HCP 9)

Johnston and Gowers [4] state that lack of training on outcome measures is a recognised barrier to the uptake of ROM, with HCPs feeling unwilling to implement them if they are unsure about how to correctly administer, score and utilise the data. Furthermore, the CYP-IAPT team [31,33] explicitly mention the importance of providing the correct infrastructure and training to facilitate ROM. Our research highlights that even when this training is offered, HCPs can be difficult to engage. Future ROM initiatives may wish to consider mandatory training sessions for HCPs to overcome this problem.

#### *Time factors*

Although administrative staff did not consider the SxS process to be particularly burdensome, they did suggest that it could be improved by requesting that patients arrive earlier for their appointment to complete SxS on a dedicated work station.

“As part of our initial appointment documentation that goes out, we would say that this is the process, please arrive fifteen minutes prior to your appointment and when they come, they are shown the work station and they input their thing” (Admin 4)

Previous research has tended to overlook the opinion of support staff when implementing ROM, however CYP-IAPT specifically mention the need to consider the implementation burden [30]. These findings suggest that adopting a whole clinic approach to ROM may facilitate the implementation process and that administration staff are willing to be involved in the process if it is streamlined to fit with existing clinic demands or if extra support is provided.

Uptake for SxS from HCPs was relatively low, which participating HCPs thought may in part stem from time concerns.

“Clinicians don't want to do something new and something that takes a bit more time” (HCP 7)

However, those HCPs who did take part noted how quick SxS was to complete and many acknowledged the importance of administrative staff being responsible for the SxS process.

“I liked that it was very easy and quick and didn't feel worried about giving it to people” (HCP 4)

“I found it quite helpful them doing it while they were waiting for appointments, which relies less on me having to remember” (HCP 2)

Young people and parents were also positive about how quick SxS was to complete.

“It took five minutes, it was fast” (Parent 10)

Time burden is perhaps one of the most significant barriers to the use of ROM [3,4,12,13] but our findings highlight that this can be alleviated by administrative staff supporting the process, provided appropriate resources and structures are put in place.

#### *Timing and location for* SxS

Confirming previous findings [3,34] and supporting the approach adopted in this study, completion of outcome measures was not viewed by HCPs as a good use of valuable session time.

“Doing outcome measures during the session, they can sometimes just get in the way of that therapeutic process a bit. I'd prefer before, perhaps before is the best option” (HCP 9)

Likewise, completion at the end of a session was not recommended due to likelihood of fatigue or socially desirable responding.

“If we ask at the end of the session, it feels a bit like they are giving us feedback about how good the session was like. And that is not a good thing because they are in front of us, they will feel under pressure to say something…. more positive than they really think” (HCP 7)

In general, young people and parents were also supportive of SxS being completed in the waiting room prior to the clinic session.

“It was just easier to do it then while you were waiting… because sometimes you could be waiting for like thirty minutes; it's a long while to wait so you could just fill that out”(Young Person 14)

However, for other young people, completing SxS in the waiting room was a concern and they reported feeling that the iPad drew unwanted attention towards them or that other people in the waiting room may be judging them.

“Well I didn't like it that much because, sometimes people were staring at it and I didn't want people to see what I was putting because it was about my issues and I don't want people to know” (Young Person 4)

Many parents acknowledged that the iPad made completing SxS in the waiting room more acceptable, feeling that the tablet offered more privacy than a paper and pen based questionnaire.

These findings demonstrate strong support from both families and HCPs that outcome measures are best completed prior to the clinic session. It appears that the use of the iPad offered some reassurance about the confidentiality of results. However, a booth in the waiting room may provide an ideal solution to completing measures in the waiting room area whilst still maintaining privacy.

Completing SxS outside the clinic settings (such as home or school visits) proved to be problematic due to difficulties in getting a network signal and remembering to take the iPad in the absence of administrative support (HCP 5 & 7). This further endorses the importance of appropriate support structures to enable HCPs to successfully administer ROM [30].

Most young people and parents were supportive of completing SxS every session, whether attending clinic on a weekly, fortnightly or monthly basis. Some families commented that they liked the routine and the brevity of questionnaire did not make it feel a burden. Families also felt that not completing it every time would undermine the purpose.

“I think yeah because it's regular, when you look at the results like this… if you miss sometimes… you're not getting a true reading are you” (Young Person 11)

To the best of our knowledge this is the first study to investigate young people and parent/carer opinions on session-by-session monitoring. It is encouraging for such systems as CYP-IAPT to observe that families do not perceive it as a burden which may help HCPs overcome some concerns with asking patients to complete multiple forms [15].

#### *Applicability of* SxS

Some HCPs suggested that SxS would not be suitable in all CAMHS settings.

“Some settings are less appropriate, for example like with younger children, they have little concentration so this wouldn't be a thing that you could use. In self harm, they mostly see people only once, a maximum of twice… but it's not always them who see them for a follow up, sometimes it's another clinician” (HCP 7)

This supports the CYP-IAPT ethos of not imposing outcome measures when it is not suitable [31] and emphasises the need to use clinical judgment.

#### *Sustaining* SxS

It was apparent that the resources provided by the research team were crucial, leaving some HCPs doubting the ability of the SxS process to work outside the support of a research project.

“Where there wasn't your [Researcher] involvement to kind of keep on top of like the data and all that kind of thing or admin ownership of that process, it just didn't work” (HCP 5)

This confirms the work of CYP-IAPT, that providing services with adequate infrastructure and support is crucial for ROM [30]. Some HCPs reported that establishing a new system of ROM may take a long while, which is likely to be the case given that integrating new procedures involves cultural and attitudinal changes [3,32].

“I know that any change is going to be very slow and it may take years sometimes. It may take a generation of clinicians… new clinicians who are more open-minded” (HCP 7)

HCPs also commented that if new systems were mandatory and routine this may help improve the procedure.

“Something that makes it more routine and less something that you've got to remember to invite people to do, would make a big difference” (HCP 4)

### Theme 3 – Helpfulness of SxS

### As administration staff were not involved in interpreting the results of the SxS, only HCPs and families were asked their opinions of the clinical utility of SxS

#### *Progress tracker*

Many HCPs and families highlighted the utility of SxS as a progress tracker, specifically mentioning the importance of being able to see the journey of change the young person was making. Parents and young people reported that seeing a visual representation of change often helped them recognise progress that they were unaware of. Some young people also commented that seeing the reports gave them hope that the intervention was working. Although this was not assessed in this study, it is possible that this ability to reflect and track progress improves patient outcomes [10].

“It showed you like, how far you've been going and like giving you motivation to try and get it up another step” (Young Person 15)

HCPs felt that reflecting on the SxS reports was particularly helpful when nearing the end of the intervention and was useful to document the journey of recovery and the change made over time from the families’ perspective. Some HCPs highlighted that they liked the simple overview of SxS and praised its ability to provide a ‘snapshot’ of progress.

“It's easy to compare things, I think, rather than flicking through notes and trying to look at how a patient was before. I think if you want a quick overview before you see a patient, especially if there's a busy clinic, it gives you an idea of how they've been over a period of time” (HCP 6)

However, other HCPs felt this was too brief to be of any clinical use, an opinion that was shared by some families.

“There's not a huge amount of clinical data which I can use, it doesn’t tell me whether you know, the medication is better or worse or whether their tics are better or worse, it's a bit too general to be able to say what's better or what's worse” (HCP 1)

“There was a lot of things you just couldn't put no detail in and so you're not getting the whole picture” (Parent 11)

This supports previous research that has shown both parents [16] and HCPs [11] feel that a questionnaire cannot encapsulate all the information about the young person. The balance between brevity and detail is one which has been recognised within CYP-IAPT [30,33] and further research on the psychometric properties and clinical utility of these measures is needed.

#### Communication and engagement

The majority of HCPs mentioned the importance of the reports as a way of engaging the patient in the session.

“I think the thing is with him, is because he can be quite hard to focus, into conversation it actually gave a chance to erm, to sort of like start with a bit of a focus. And to start with a point of reflection” (HCP 5)

Some HCPs also described that the report often helped expand discussion points which was particularly useful with teenagers or young people who struggled to communicate verbally.

“You sometimes don't give a lot of verbal feedback, sometimes the [SxS] can be a different way of doing it….communicating how things are” (HCP 3)

The idea of outcome measures aiding the session is one that is typically overlooked in research, with a tendency to focus on the reliability and utility of the data itself. Additionally, HCPs commented on how comparing the reports of the young people and parents often opened up channels of communication within the family.

“It also gives a comparable opinion of the young person and the parents or care-giver and that doesn't always happen in the room” (HCP 7)

“I think it helped us to understand not only what you’re thinking at the time but what your child is thinking and if you’re thinking the same thing” (Parent 3)

However, other HCPs reported that SxS was sometimes a barrier to engaging the young person in the session, which hindered the flow of their normal sessions. For instance, some noted difficulties in being able to view the iPad in sessions.

“You've got to read it and then they sit there in silence and then you've got somebody who has got tics or has ADHD and they start kicking at blocks, the door, shouting at you or clicking or throwing something round the room” (HCP 1)

If future systems can link with NHS Trust IT, it ought to be possible for the HCP to have access to this information before the young person enters the clinic room which may help overcome this barrier.

Families that had not been shown their report by the clinician tended to have a more negative view on the usefulness of SxS, supporting the assertion that ROM is only valued if data is fed back [3,16].

“I think there's not really much point in sitting and doing a questionnaire that isn't being used to help you. Because it's obviously is there to help, so, if it's not getting used to help you, what's the point of it being there?” (Young Person 8)

#### Improvements to reports

Both HCPs and families were divided on whether they found the reports easy to understand. Many HCPs and families considered the reports were easy to understand without instruction and appreciated the visual depiction. However, some HCPs reported that the graphing system was not simple enough and some families commented that it was not always obvious how to interpret the graphs.

“At first glance you're not really sure whether you're looking for a bigger bar or a smaller bar” (Parent 4)

Clearly no report system is going to satisfy everyone, but our findings support the use of clear outputs that are instantly understandable and intuitive. Perhaps a combination of visual and numeric information would provide a suitable compromise.

#### *Reflection*

Regardless of seeing the outputs, many families commented that completing the questions was a helpful process in its own right. Parents and young people reported that it reminded them to ask specific questions to their HCP and that it gave them an opportunity to reflect on how they had been since their last session. As a result of this, families reported feeling more prepared for their session and felt more able to assess their problems and the current situation. This further supports the utility of ROM as part of the therapeutic process.

“Prompting people, to take stock of an analysis of the past, what is happening right now based on the past, projecting into the future what could happen…in order to change what could happen… that’s what we need to do” (Parent 9)

### Theme 4 – attitudes towards technology

As only the HCPs and families were responsible for completing the SxS and the associated reports, only these groups were asked about completing measures electronically.

The use of an iPad to complete the SxS questionnaire divided HCPs and families. In general, HCPs were very supportive of an electronic questionnaire. They attributed the ease and brevity of the questionnaire to the fact it was completed electronically. Most prominently, HCPs felt the iPad was of particular interest to the young people.

“I think it feels more… valuing of them, that we are giving them a piece of technology to use and the iPad is quite… a desirable thing isn't it, so it feels nicer to give that out than to give someone a really rubbish photocopy” (HCP 4)

In support of this, Truman et al. [35] piloted an electronic version of the SDQ and found that patients considered the computer version more interesting and easier to complete which also led to greater inter-rater reliability and internal consistency than the paper-based version, indicating that computerised ROM may also help improve validity and reliability.

Some HCPs specifically mentioned that the young people’s positivity towards the iPad and their willingness to use it each time was a specific motivating factor for their use of SxS. Many young people commented that completing the questionnaire on the iPad, was ”*more fun*”, ”*cool*” and less effort than a paper-based version. However, some families’ reported they would have preferred to complete the questionnaire by pen and paper. Support for the use of a paper-based SxS mainly stemmed from technical issues that they had experienced when using the iPad.

“I'd say [I’d prefer to use] paper but only because, times that I did use it, it didn't seem to follow that well, we tried and save the screen and whatever, I can't even remember what we had to do and it wouldn't… it kept crashing as well” (Parent 4)

It is likely that both HCPs and patients would become disengaged with a system that is unreliable to run, so support from internal Trust IT systems would be imperative to the success of any computer-based ROM system.

### **Theme 5 -** SxS **Content**

As administration staff were not responsible for reading the SxS report or questions, only HCPs and families were asked about the content of the measure.

Families often reported that they were pleased with the brevity of SxS and found the questions easy to understand. However, several families mentioned difficulties with being able to encapsulate the daily or weekly fluctuations they experienced.

“Some of the questions, they are just straight answers and sometimes you can't answer, give a straight answer… because they are up one minute down and then up the next” (Parent 11)

The question that assesses aspiration of hope for the future was considered by HCPs to reflect families’ faith in the intervention they were receiving and the confidence they had in their HCP to help them progress.

“I haven't found that [Hope] in any other outcome measures. It's sort of a projection to the future, which reflects how much hope the person has about change, how much trust in their ability to change, in your ability to help them improve so…..it tells you something about the attitude and perspective of the young person that otherwise you wouldn't know. You usually don't ask this. And it's an important thing that determines how things will be” (HCP 7)

However, families often reported this question as being too difficult to answer.

“How are you going to be next month…I mean it's like looking at let me get my crystal ball out and have a look” (Parent 7)

Both HCPs and families mentioned the importance of having a question which assesses therapeutic alliance, and felt this was something currently missing from SxS.

“I think an important question is about therapeutic alliance, or the therapeutic process… would be an important thing to capture on a session by session basis” (HCP 5)

The CYP-IAPT recommends the use of the Session Rating Scale (SRS; [36]) or the short feedback questionnaire (http://www.IAPT.nhs.uk) at the end of each session as a measure of the therapeutic relationship. Our findings show that both HCPs and families are supportive of a measure that assesses this concept.

Young people and parents positively commented on the response boxes which allowed them to select options which reflected small progress, such as ”a little better” as well as ”much better”. However, several families felt they would have preferred a sliding scale of 1–10 to further increment their journey and the opportunity to write free text, a finding also noted by Moran et al. [16].

“..Well you can't answers questions like that in a box… No there's no elaboration” (Young Person 9)

By gathering the perspectives of HCPs, families and administrative staff we have provided a novel and holistic evaluation of current attitudes towards session-by-session ROM in CAMHS using an electronic-based questionnaire. Our findings identify a variety of advantages and disadvantages to the use of ROM. Many of our findings support previous research investigating attitudes towards ROM [4,11,15,16], as well as points raised by the CYP-IAPT team [30-32]. In the main, the young people and parents/carers in this study were supportive of completing measures prior to clinic sessions, with administrative staff taking ownership of the process seeming a promising model of delivery. Families were supportive of questions that were meaningful to the individual and the fact that SxS was quick and easy to complete and could facilitate real-time feedback. Negatives issues related to problems with the technology, data that were not perceived to be clinically useful or able to capture enough detail, disruptions to the therapeutic sessions and some difficulties engaging HCPs. We particularly noted difficulties in engaging HCPs in ROM. Although previous work has noted lack of training as barrier to ROM [4], our findings demonstrated that even if training is offered, clinicians are unwilling to participate in training or ROM completion. On the basis of this, we suggest that training on ROM may need to be mandatory. The current findings indicate that session-by-session measurement would be welcomed in CAMHS if appropriate administrative and technical support was available and the resulting data were be fed back to both the HCP and patient in a manner that was both clinically meaningful and easy to understand. Additionally, promoting a ‘clinic-wide’ adoption of ROM in which a standard procedure is operated for each case would facilitate the adoption of ROM in routine clinical practice.

### Strengths and limitations

To the best of our knowledge this is the first research that investigates opinion of session-by-session outcome measures within CAMHS. Furthermore, our research provides an under-investigated insight into clinicians’, caregivers’ and young people’s opinion of incorporating new technologies to improve healthcare services. Our research is strengthened by the inclusion of all users of outcome measures, including the opinions of young people, parents/carers and administrative staff, who are often overlooked. However, our findings need to be considered in light of a limitation of setting the study in one NHS Trust; as such, caution should be taken when generalising the findings to other clinics located in different geographical regions. Although a strength of our research is the inclusion of HCPs of different professional backgrounds working within CAMHS and the inclusion of a range of young people with a variety of mental health diagnoses, participation in the study was optional. It may be that the HCPs or young people/parents/carers who participated in SxS were particularly motivated or interested in the use of outcome measures. Some of our findings are likely to be specific to SxS; as such, further research on other session-by-session measures is needed. Clearly, the next step would be to implement a service or clinic-wide use of session-by-session outcome measures to assess the feasibility of this system of outcome measurement as part of routine clinical practice.

## Conclusions

The findings provide a valuable and under-researched insight into how clinical staff and patients view the use of electronic session-by-session measurement within CAMHS. In doing so, we specifically highlight the need for appropriate infrastructure, support and training to establish an effective system of ROM. Our findings indicate that session-by-session monitoring would be welcomed by HCPs, families and administrative staff alike if the system is not too onerous and the information is clinically useful. We particularly noted initial clinician resistance to ROM and advocate the need for mandatory training on outcome measures to improve clinician understanding. Although opinions on technology were mixed, we advocate a system that is quick and simple to use, that does not take time away from the clinic session and involves the co-operation of administrative support staff to safeguard clinician time. This is likely to be facilitated by a clinic-wide standard procedure for ROM.

### Ethical & R & D approval

Ethical approval was granted by the Nottinghamshire Ethics Committee and R&D approval was obtained from Nottinghamshire Healthcare NHS Trust (NHCT).

## Competing interests

RG is owner of Youthinmind Ltd which produces no-cost and low-cost websites related to the SDQ and SDQ-related session by session monitoring.

## Authors’ contributions

CLH and SP created interview schedules. CLH conducted the interviews and analysed the results. KN assisted in data analysis. The data collection, analysis and write-up were guided by JT. All authors contributed to the interpretation of the data and the study write-up. CLH drafted the manuscript and JT, MM, MMa, SP, KN, RG, KS and CH revised it critically for important intellectual content.

## Pre-publication history

The pre-publication history for this paper can be accessed here:

http://www.biomedcentral.com/1471-244X/14/113/prepub

## Supplementary Material

Additional file 1 Interview schedule – admin. Click here for file

Additional file 2 Interview schedule – clinician. Click here for file

Additional file 3 Interview schedule – young person & parent. Click here for file
