# Supplementary material for: A qualitative process evaluation of electronic session-by-session outcome measurement in child and adolescent mental health services
Source: BMC Psychiatry. 2014 Apr 15;14:113. doi: 10.1186/1471-244X-14-113 (PMC4021403; doi:10.1186/1471-244X-14-113)
Supplement: Additional file 1 — Interview schedule – admin. [file 1471-244X-14-113-S1.docx]

**INTERVIEW SCHEDULE – ADMIN**

**SXS**

**Introduction**

Researcher reminds participant on the study and goes through ethical procedure.

**General Background Questions**

1. Can you give me a little information on your professional background

- How long have you worked in CAMHS.
- training
- How long have you been a medical secretary (or whatever role)
- How long have you been in Mansfield/TW/QMC

**SXS questions**

1. How did you come to be involved in ROMPT

- Who approached you?
- What did they tell you?

1. How did you feel about being involved?

- Know what was expected of you?
- Training
- Concerns on time
- Concerns with ipad

1. How do you feel about it now?

- Has it run better / worse than you thought?
- Have you had any problems?

1. What helped you use it?

- Other members of admin support. Clinician support. Manual. Researcher. Time. Young Person/parent support

1. What hindered you using it?

- Negative attitude from YP / clinician / other admin. Lack of time. Ipad not working

1. Do you think SXS is running well in this clinic?

- Why or why not?

1. Do you know why we are using SXS?

- What it is supposed to help with? Whats the point in it?

1. Did it help or interfere with you carrying out your normal duties?

**Future of SXS**

1. Would you recommend it was used routinely in clinics?

- Why?

1. Would you recommend it being used routinely in other clinics? (**too similar to Q8?)**

- Why?

1. If SXS was to continue as part of routine clinical practice what suggestions would you make to improve the process?
