# Supplementary material for: A qualitative process evaluation of electronic session-by-session outcome measurement in child and adolescent mental health services
Source: BMC Psychiatry. 2014 Apr 15;14:113. doi: 10.1186/1471-244X-14-113 (PMC4021403; doi:10.1186/1471-244X-14-113)
Supplement: Additional file 2 — Interview schedule – clinician. [file 1471-244X-14-113-S2.docx]

**INTERVIEW SCHEDULE – CLINICIAN**

**introduction**

Researcher reminds participant on the study and goes through ethical procedure.

**General Background Questions**

1. Can you give me a little information on your professional background

- How long have you worked in CAMHS.
- training
- Profession
- How long have you been in Mansfield/TW/QMC

**Outcome Measures Questions**

1. What is your involvement with routine outcome measures in general?

- Why do you use them? Daily for treatment, for commissioning, management? How frequently do you use them?

1. Is your involvement specifically related to CAMHS, or have you had previous experience of ROMs?
2. How important are ROMS to your own personal practice?
3. Why do you use routine outcome measures?

- Is it a requirement? Would they do it if not?

**General SXS questions**

1. Do you know why we are using SXS?

- What it is supposed to help with? Whats the point in it?

1. Did it help or interfere with you carrying out your normal duties?
2. How many times have you used SXS?

- Rough idea – lots, only a few.

1. What cases did you use SXS with?

- Why these cases?

1. Why did you decide to be involved in SXS?

- Like ROM
- Manager pressure
- Wanted to help

1. How did you feel about being involved?

- Know what was expected of you?
- Training
- Concerns on time
- Concerns with ipad

1. How do you feel about it now?

- Has it run better / worse than you thought?
- Have you had any problems?

1. What helped you use it?

- Members of admin support. Other clinician support. Manual. Researcher. Time. Young Person/parent support

1. What hindered you using it?

- Negative attitude from YP / other admin. Lack of time. Ipad not working

1. What did you think about completing SXS in waiting room?

- Work well? Prefer in session? Why?

1. Do you think SXS is running well in this clinic?

- Why or why not?

**SXS questions & output Questions**

1. Do you feel it asks the right questions?

- Which ones did you like? Not like?
- What was missing?

1. What did you think about the report/graph/bar charts?

- What was good. What was bad?
- Were they interesting? Helpful? Unhelpful?

1. How could we improve the report/graph/bar charts?
2. Did the change illustrated in the graph agree with your clinical opinion of change?
3. Did you discuss the output with your patient?

- Always? Sometimes?
- Was this helpful? Why?
- In which cases?

1. Did you find it interesting to compare answers of the parent and child?

- Why?

**Future of SXS**

1. Would you recommend it was used routinely in clinics?

-Why?

1. Would you recommend it being used routinely in other clinics?

-Why?

1. If SXS was to continue as part of routine clinical practice what suggestions would you make to improve the process?
