# Supplementary material for: A qualitative process evaluation of electronic session-by-session outcome measurement in child and adolescent mental health services
Source: BMC Psychiatry. 2014 Apr 15;14:113. doi: 10.1186/1471-244X-14-113 (PMC4021403; doi:10.1186/1471-244X-14-113)
Supplement: Additional file 3 — Interview schedule – young person & parent. [file 1471-244X-14-113-S3.docx]

**INTERVIEW SCHEDULE – YOUNG PERSON & PARENT**

**SXS**

**My introduction**

Researcher reminds participant on the study and goes through ethical procedure.

**General Background Questions**

1. Let’s start by getting to know you a bit. Can you tell me a little about yourself? Such as hobbies?

- Hobbies / where go to school/college / likes and dislikes (Amend for parent/child)

**SXS Procedure questions**

1. How did you come to be using SXS?
2. Why did you choose to complete SXS?

- Didn’t feel they had a choice? Wanted too? Interested?

1. Do you know why we are using SXS?

- What it is supposed to help with? Whats the point in it?

1. How many times have you used SXS?

- Rough idea – lots, only a few.
- Every time you attended clinic or only sometimes? Why?

1. What did you think about completing SXS on the iPad?

- Easy to use?
- Comfort with technology? Used iPad before?
- Support admin staff? Clinicians? Manual?

1. What did you think about completing SXS in waiting room?

- Work well? Prefer in session? Why?
- Rushed? Helped boredom?

**SXS Questions**

1. Was it easy to complete the questions?

- Understood questions?
- Response boxes?

1. Do you feel it asks the right questions?

- Which ones did you like? Not like?
- What was missing?

**Output Questions**

1. What did you think about the report/graph/bar charts?

- What was good. What was bad?
- Were they interesting? Helpful? Unhelpful?

1. How could we improve the report/graph/bar charts?
2. Did your clinician discuss the output with you?

- Always? Sometimes?
- Was this helpful? Why?

1. If Mum or Dad completed as well – did you find it interesting to compare answers?

- Why?
- Did this help with any issues in understanding each other? Getting views across?

1. Did you feel that the graph was correct?

- Did it show the change that you felt

**Future SXS questions**

1. Overall, have you enjoyed using SXS?

- If you could go back to when you said ‘yes’ would you still say yes?

1. Would you recommend using SXS to other people?

- Who?
- Why?

1. Do you think SXS should be completed at every session?

- Every other? Most sessions? All? Why?

1. How could we improve SXS in the future?
